# Successful Treatment of Coexisting Paraganglioma of the Retroperitoneum and Urinary Bladder by Intermediate-Dose 131I-MIBG Therapy: A Case Report

**DOI:** 10.1097/MD.0000000000001686

**Published:** 2015-10-16

**Authors:** Yi Cai, Han-Zhong LI, Yu-Shi Zhang

**Affiliations:** From the Department of Urology, Peking Union Medical College Hospital, Chinese Academy of Medical Sciences and Peking Union Medical College, Beijing 100730, China.

## Abstract

Paragangliomas (PGLs) are rare neuroendocrine neoplasms of the autonomic nervous system dispersed from the skull base to the pelvic floor. We reported an extremely rare case with coexisting PGL of the retroperitoneum and urinary bladder. The patient complained of episodes of severe headache and palpitation during micturition, with a 1-year history of uncontrolled hypertension. The ^131^I-metaiodobenzylguanidine (^131^I-MIBG) scintigraphy revealed high uptake in both of the retroperitoneum and urinary bladder mass accompanied with increasing 24 hour urinary norepinephrine and epinephrine. The patient received 6 doses of high-specific activity ^131^I-MIBG from October 2009 to April 2015, each treatment dose was 400 mCi. The patient got symptomatic, hormonal, and radiographic tumor complete response without life-threatening adverse events.

Intermediate-dose ^131^I-MIBG therapy may be an alternative choice for patients with multicentric PGLs with positive MIBG scintigraphy.

## Abstract 2

Supplemental Digital Content is available in the text

## INTRODUCTION

Paragangliomas (PGLs) are rare neuroendocrine neoplasms of the autonomic nervous system (2–8 per million populations), a diffuse neuroendocrine system dispersed from the skull base to the pelvic floor.^1^ Urinary bladder PGL constitutes only 0.06% of all bladder tumors and 6% of all extra-adrenal PGLs.^2^ There are few reports about concurrent PGLs of the retroperitoneum and urinary bladder.^3^ Although surgical resection stands for the first treatment option for PGLs, radionuclide ^131^I-metaiodobenzylguanidine (^131^I-MIBG) therapy has been widely used for patients with malignant PGLs to cure or control inoperable tumor since the 1980s.^4^ But few cases can get complete response (CR) by low or intermediate-dose ^131^I-MIBG therapy.^5^ Here, we reported an extremely rare case of concurrent PGL of the retroperitoneum and urinary bladder. After received 6-times intermediate-dose ^131^I-MIBG therapy, the patient got symptomatic, hormonal, and radiographic tumor CR without life-threatening adverse events.

## CASE REPORT

**FIGURE 1:**
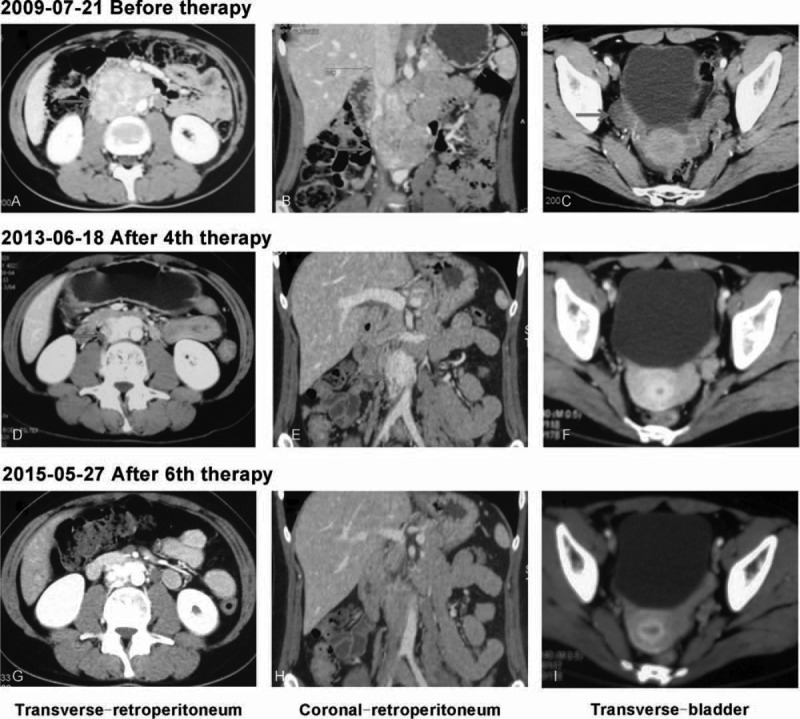
Abdominal computed tomography showed lesion of the retroperitoneum and urinary bladder before and after ^131^I-metaiodobenzylguanidine (^131^I-MIBG) therapy. (A–C) Before ^131^I-MIBG therapy; (D–F) after 4 course ^131^I-MIBG therapy; and (G–I) after 6 course ^131^I-MIBG therapy.

**FIGURE 2:**
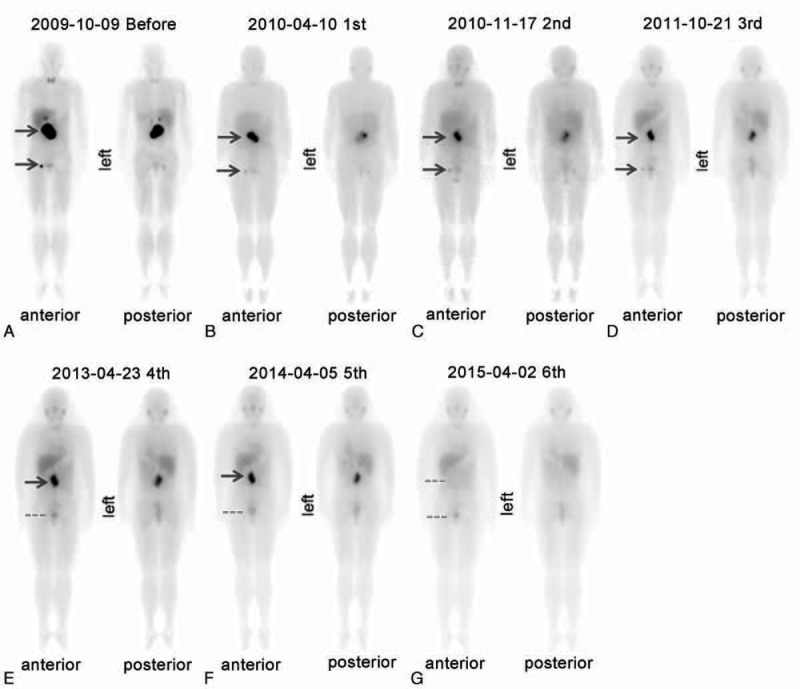
^131^I-metaiodobenzylguanidine (^131^I-MIBG) scintigraphy revealed mass of the retroperitoneum and urinary bladder before and after ^131^I-MIBG therapy.

An 18-year-old woman was referred to our hospital in July 2009 for a 1-year history of uncontrolled hypertension (BP_max_ 185/120 mmHg). The patient was treated with β-blocker, calcium channel blocker, and angiotensin II receptor blocker, however, without satisfactory effect. The patient also complained of episodes of severe headache and palpitation during micturition, without history of lower urinary tract symptoms or hematuria. The physical examination and routine laboratory investigations were normal. The patient's height is 163 cm while the weight is 53 kg. On ultrasound abdomen 9.2 × 3.9 cm low echo-level lesion was seen in the retroperitoneum next to aorta in front of spine and 4.1 × 2.3 cm low echo-level lesion was seen in right posterior wall of the bladder. Computed tomography (CT) abdomen revealed 8.5 × 6.1 × 3.7 cm well capsulated lesion in portocaval region with increased enhancement in arterial phase and relative washout in portovenous phase. Part of portal vein, abdominal aorta, and renal artery were wrapped by the lesion (Figure 1A, B). A 3.5 × 2.1 × 1.3 cm similar enhancing lesion in right posterior bladder wall was present (Figure 1C). Twenty-four hour urinary norepinephrine was 465.45 μg/24 hour (normal: 16.69–40.65 μg/24 hour), urine epinephrine 26.76 μg/24 hour (normal: 1.74–6.42 μg/24 hour), while dopamine 124.5 μg/24 hour (normal: 20.93–330.59 μg/24 hour) within normal limits. Then, ^131^I-MIBG scintigraphy was performed which revealed high uptake only in both of the retroperitoneum and urinary bladder mass, but not in the adrenal glands or elsewhere (Figure 2A). Then, the diagnosis of concurrent PGL of the retroperitoneum and urinary bladder was made.

**FIGURE 3:**
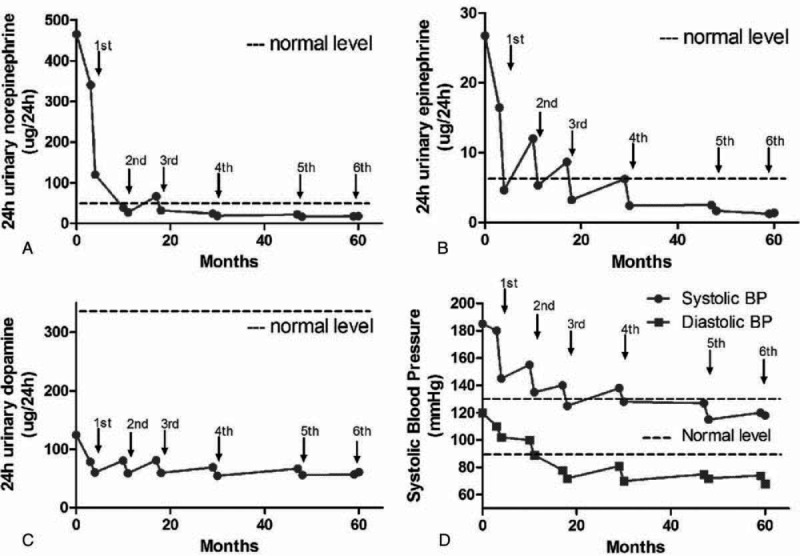
(A–C) Twenty-four hour urinary norepinephrine decreased after ^131^I-metaiodobenzylguanidine (^131^I-MIBG) therapy. (D) Systolic and diastolic blood pressure decreased normal level after ^131^I-MIBG radiotherapy in the patient.

As the patient and her parents refused to be surgically excised, she was given the intermediate-dose ^131^I-MIBG therapy for the young patient without any basic diseases. ^131^I-MIBG therapy was approved by the Institutional Review Board. Written informed consent was also obtained before therapy. The patient received 6 doses of high-specific activity ^131^I-MIBG from October 2009 to April 2015, each treatment dose was 400 mCi. ^131^I-MIBG was administered as a slow intravenous infusion over a period of 1 hour. Thyroid uptake of free radioiodine was blocked either by potassium iodide 200 mg/day plus levothyroxine 100 μg/day for 72 hours prior to and 14 days after therapy. Blood pressure and heart rate were monitored during ^131^I-MIBG infusion. Posttherapeutic ^131^I-MIBG imaging was performed 4 to 6 days after therapy. Toxicity was evaluated by extensive laboratory tests including complete blood count, liver function, and thyroid function tests. Twenty-four hour urinary catecholamines levels were measured 24 hours prior to and 7 days after each course therapy (Figure 3A–C). CT abdomen was used to assess the radiological tumor response within 3–6 months after the each dose of ^131^I-MIBG. The symptomatic, hormonal, and radiological tumor response, according to WHO criteria, assessed within 6 months after the each dose of ^131^I-MIBG. CR for symptomatic, hormonal, and radiographic tumor were defined as complete normalization for symptomatic response, catecholamine levels, and complete regression of radiological abnormalities and also a negative diagnostic ^131^I-MIBG scan. Partial response (PR) for symptomatic, hormonal, and radiographic tumor were defined as subjective decrease in intensity and frequency of symptoms, 50% or greater reduction from pretherapy level of hormones and 50% or greater reduction of all measurable tumor. After 4 course of ^131^I-MIBG therapy, the patient got symptomatic and hormonal CR. The lesion of urinary bladder was disappeared with CT (Figure 1E) and ^131^I-MIBG scan (Figure 2E), while the focus of retroperitoneum reduced more than 50% (PR) (CT scan: 2.3 × 1.8 × 1.3 cm) (Figure 1C, D). After 6 course intermediate-dose course of ^131^I-MIBG therapy, the patient got radiographic tumor CR (Figure 1G–I and Figure 2G). We monitor the myelosuppression and other toxicity closely by laboratory tests including complete blood count and liver function tests. The Supplemental Table 1, http://links.lww.com/MD/A451, contains complete blood count, liver function, and thyroid function tests before and after treatment. There are no life-threatening adverse events were observed.

## DISCUSSION

PGLs are derived from either sympathetic chromaffin tissue extra-adrenal locations or parasympathetic tissue of the head and neck.^1^ Although most PGLs are solitary and typically arise sporadically, they can be multicentric or hereditary. Previous studies reported few case of PGL of the retroperitoneum and urinary bladder.^3^ Complete surgical resection represents the curative treatment option for PGLs. However, benefits and potential risks of all treatment options may be taken into consideration for every individual patient. In this case, the patient and her parents refuse to accept surgery as an option for potential mortality of the surgical procedure of PGLs. In some patients for whom surgery may not be the best option, tumors in accessible locations can be addressed by radiofrequency ablation.^6^ Chemotherapy is an option for patients with malignant PGL. Huang et al^7^ reported a combination chemotherapy with cyclophosphamide, vincristine, and dacarbazine produced PR or CR in 10 of the 18 patients. In addition, antiangiogenic therapy with sunitinib may prove useful in the treatment of malignant PGL.^8^

^131^I-MIBG has been used for patients with malignant pheochromocytoma and PGL to cure or control inoperable tumors since 1983. Not all patients with PGL are eligible for MIBG therapy, as it depends on whether the tumors exhibit adequate take up of the radiopharmaceutical after intravenous administration. For patients with positive MIBG scintigraphy, MIBG therapy can be a valuable treatment modality. The ^131^I-MIBG therapy protocols can be categorized into 3 types based on the dose: low-dose (80–200 mCi/person/session), intermediate-dose (up to 500 mCi/person/session), and high-dose therapy (11.89–18.10 mCi/kg/session).^9^ Previous studies showed that high-dose regimens could obtain relatively good therapeutic effect but also higher risk for side effect of bone marrow suppression.^10^ The intermediate-dose ^131^I-MIBG therapy requires multiple courses, but there was no consensus as to how many times and few patients could get CR. Few patients can get CR from MIBG treatment, with the highest reported rates only about 15%.^11^ A recently systematic review of published studies on ^131^I-MIBG therapy showed that only 3% had a CR in 243 patients.^5^ However, parts of patients can benefit from PR, lower biochemical levels, and reduced symptoms.

In our case, the patient got symptomatic, hormonal, and radiographic tumor CR after 6 course of ^131^I-therapy. There were no life-threatening adverse events observed. In all, the coexisting PGL of the retroperitoneum and urinary bladder are extremely rare tumors and this patient in the present case was the first cases got CR after repeated intermediate-dose ^131^I-MIBG treatment.

## CONCLUSION

We reported an extremely rare case with coexisting PGL of the retroperitoneum and urinary bladder. After 6 course intermediate-dose course of ^131^I-MIBG therapy, the patient got symptomatic, hormonal, and radiographic tumor CR without life-threatening adverse events. Intermediate-dose ^131^I-MIBG therapy may be an alternative choice for patients with multicentric PGLs.

## References

[1] Martucci VL Pacak K Pheochromocytoma and paraganglioma: diagnosis, genetics, management, and treatment. *Curr Probl Cancer* 2014; 38:7–41. 24636754 10.1016/j.currproblcancer.2014.01.001 PMC3992879

[2] Bhalani SM Casalino DD Manvar AM Paraganglioma of the bladder. *J Urol* 2011; 186:279–280. 21600612 10.1016/j.juro.2011.04.032

[3] Verma A Non-functional paraganglioma of retroperitoneum mimicking pancreatic mass with concurrent urinary bladder paraganglioma: an extremely rare entity. *J Clin Diagn Res* 2015; 9:XD1–XD09. 10.7860/JCDR/2015/11156.5570 PMC4378794 25859512

[4] Sisson JC Shapiro B Beierwaltes WH Radiopharmaceutical treatment of malignant pheochromocytoma. *J Nucl Med* 1984; 25:197–206. 6726430

[5] Van Hulsteijn LT Niemeijer ND Dekkers OM 131I-MIBG therapy for malignant paraganglioma and phaeochromocytoma: systematic review and meta-analysis. *Clin Endocrinol* 2014; 80:487–501. 10.1111/cen.12341 24118038

[6] Pacak K Fojo T Goldstein DS Radiofrequency ablation: a novel approach for treatment of metastatic pheochromocytoma. *J Natl Cancer Inst* 2001; 93:648–649. 11309443 10.1093/jnci/93.8.648 PMC2386878

[7] Huang H Abraham J Hung E Treatment of malignant pheochromocytoma/paraganglioma with cyclophosphamide, vincristine, and dacarbazine: recommendation from a 22-year follow-up of 18 patients. *Cancer* 2008; 113:2020-8. 18780317 10.1002/cncr.23812 PMC9094399

[8] Joshua AM Ezzat S Asa SL Rationale and evidence for sunitinib in the treatment of malignant paraganglioma/pheochromocytoma. *J Clin Endocrinol Metab* 2009; 94:5–9. 19001511 10.1210/jc.2008-1836

[9] Yoshinaga K Oriuchi N Wakabayashi H Effects and safety of 131I-metaiodobenzylguanidine (MIBG) radiotherapy in malignant neuroendocrine tumors: results from a multicenter observational registry. *Endocr J* 2014; 61:1171–1180. 25214026 10.1507/endocrj.EJ14-0211

[10] Gonias S Shiboski S Fitzgerald P Phase II study of high-dose [131I]metaiodobenzylguanidine therapy for patients with metastatic pheochromocytoma and paraganglioma. *J Clin Oncol* 2009; 27:4162–4168. 19636009 10.1200/JCO.2008.21.3496 PMC2734428

[11] Carrasquillo JA Pandit-Taskar N Chen CC Radionuclide therapy of adrenal tumors. *J Surg Oncol* 2012; 106:632–642. 22718415 10.1002/jso.23196

